# Source Space Analysis of Event-Related Dynamic Reorganization of Brain Networks

**DOI:** 10.1155/2012/452503

**Published:** 2012-10-11

**Authors:** Andreas A. Ioannides, Stavros I. Dimitriadis, George A. Saridis, Marotesa Voultsidou, Vahe Poghosyan, Lichan Liu, Nikolaos A. Laskaris

**Affiliations:** ^1^Laboratory for Human Brain Dynamics, AAI Scientific Cultural Services Ltd., Office 501 Galaxias Center, 33 Arch. Makarios III Avenue, 1065 Nicosia, Cyprus; ^2^Artificial Intelligence & Information Analysis Laboratory, Department of Informatics, Aristotle University, 54124 Thessaloniki, Greece

## Abstract

How the brain works is nowadays synonymous with how different parts of the brain work together and the derivation of mathematical descriptions for the functional connectivity patterns that can be objectively derived from data of different neuroimaging techniques. In most cases static networks are studied, often relying on resting state recordings. Here, we present a quantitative study of dynamic reconfiguration of connectivity for event-related experiments. Our motivation is the development of a methodology that can be used for personalized monitoring of brain activity. In line with this motivation, we use data with visual stimuli from a typical subject that participated in different experiments that were previously analyzed with traditional methods. The earlier studies identified well-defined changes in specific brain areas at specific latencies related to attention, properties of stimuli, and tasks demands. Using a recently introduced methodology, we track the event-related changes in network organization, at source space level, thus providing a more global and complete view of the stages of processing associated with the regional changes in activity. The results suggest the time evolving modularity as an additional brain code that is accessible with noninvasive means and hence available for personalized monitoring and clinical applications.

## 1. Introduction

Even the simplest of tasks, be it purely cognitive, purely muscular, or a combination of the two involves coordinated activity in distinct brain areas distributed across the cortical mantle and deep brain nuclei. The coordination however is full of redundancy so that an identical goal, for example, a movement of a finger or an eye, the perception of a simple figure or a letter, can be accomplished in any of number of possible ways [1]; only in pathology the repetition of an identical task involves a rigid repetition of a fixed sequence of brain activations. The apparent orderly progression of activity that the averaged (over many trials and subjects) electroencephalography (EEG) and magnetoencephalography (MEG) data reveal is a mirage of a sandwich of histories [2]. There is however much useful information in this mirage, primarily the identification of key brain areas and points in time. In terms of cortical networks the analysis of gross measures like the average signal elicited by a large number of identical stimuli can reveal key nodes in the network that play critical role in the task and points in time when stages of processing reach a climax or are completed. Careful experimental design can then help identify the stages of processing supporting the perception of a specific type of stimuli or how they are underpinning a specific task.

Here, we adapt a dynamic graph theoretical formalism [3, 4] to the study of real-time regional brain activations derived from tomographic source analysis of MEG signals [5]. In line with many recent studies we use phase coupling to describe quantitatively the stimulus- and task-related synchronization (as a putative mechanism for long-range integration) of brain responses. We use single trial tomographic estimates of brain activity to statistically identify the key individual brain regions and derive dynamic brain networks and their graph theoretical properties. The methodology allows us to generalize the viewpoint of isolated regional changes in brain activity to the wider framework of quantifiable changes in network properties and organization. We are specifically interested in personalized monitoring of brain function and therefore apply the methodology to the single trial MEG data from individual subjects in two different sets of experiments. The first set of experiments was designed to examine the effect of attention on the earliest sensory processing stages in the brain. In our earlier study of regional time courses we found that spatial attention modulates the initial feedforward response in the primary visual cortex beginning at ~55 ms [6]. The second set of experiments focuses on the use of letter and pseudoletters as elements for category selection. The earlier analysis of neural activity in individual brain regions revealed key roles of cuneus and fusiform gyrus (FG) in this task [7]. 

Using methodologies and algorithms from the well-established branch of graph theory, the multidisciplinary approach of *complex-network* analysis characterizes a wide range of naturally occurring systems by quantifying the topologies of their network representations [8–10]. A wide range of methods have been proposed to characterize the rich anatomical and functional connectivity patterns of the brain using signals from modern brain-imaging techniques, such as diffusion MRI, functional MRI (fMRI), and EEG/MEG [11–14]. In retrospect, the description of the anatomical and functional organization of the brain as complex networks seems almost unavoidable and the question is which of the many different ways is more appropriate. The vast majority of previous studies have relied on analyzing the topological properties of static graphs, where nodes correspond to distinct brain regions and link to pairwise associations among them. The links in these graphs remain unaltered over time. Whereas this approach is reasonable for anatomical connectivity, it fails to account for the labile and highly dynamic nature of brain activity, particularly when signals from fast-recording modalities of EEG/MEG are used [15]. It is widely accepted that perceptually and behaviorally relevant events are reflected in the changes of neural activity in large-scale distributed neuronal networks [16]. However, it is much less clear how these networks are organized dynamically. This is due to the inherent difficulty in extracting information, from multiple recording sites, about the various processes participating in a particular cognitive task. The situation is further complicated by the fact that not only the state of any particular cortical area, but also the relations between cortical areas can shift rapidly on a time scale of tens of milliseconds [17, 18].

Recently a few studies have appeared focusing on the evolution of connectivity pattern as this is estimated from multichannel recordings. These studies use a moving time-window to estimate distinct connectivity graphs from the enclosed signal-segments, and by employing a topological descriptor (i.e., network metric), they derive as output a timeseries reflecting the event-related network self-organization. In most network evolution studies the connectivity is computed from the analysis of the raw signals of individual EEG/MEG sensors [19, 20]. However, a sensor based description of connectivity does not necessarily relate in an obvious way to the actual functional connectivity in the brain. For EEG, volume conduction effects and the high resistivity of the skull make the signal of each EEG electrode sensitive to a large number of brain areas. In the case of MEG, the signal of magnetometers and axial gradiometers are also influenced by a number of generators and in addition the same generator in the brain gives rise to strong contributions in different MEG sensors. The connectivity pattern computed directly over EEG/MEG sensors must therefore be interpreted with caution.

As a remedy, neuronal interactions can be studied based on signals resulting from source reconstruction [15, 21–23]. It is possible to compute the connectivity pattern from actual source activity estimates derived from the average signal of multichannel recordings of either EEG [24] or MEG [25]. To fully capture the dynamic changes in event-related connectivity, it is necessary to use single trial tomographic solutions, but this has so far been attempted for only a small number of areas [26, 27]. The results from studies using only a small fraction of the network nodes do not necessarily reflect large scale changes in network organization. Here, we combine the potential of time-varying network-analysis [3, 4] with the power of tomographic source reconstruction derived either from single trial MEG signals or many averages of few trials. The analysis allows us to examine visual response mechanisms under the innovative perspective of network reconfiguration dynamics. 

## 2. Methods

### 2.1. Quantifying Functional Brain Connectivity

#### 2.1.1. Time-Varying Functional Connectivity

Complex networks are characterized by recurring patterns, motifs, and abrupt changes in network organization that demand the refinement of existing methods and the development of new ones [10]. In an effort to accommodate the need for a dynamic description of functional brain connectivity, we have developed a network analysis framework for quasi-instantaneous estimates of connectivity patterns. In each step of the analysis, functional coupling measures capture a snapshot of connectivity within a window that encompasses only a small segment of the signals. The window is then moved forward by a fixed step to track the time evolution of the computed quantities. The method can be applied to broadband signals or to signals filtered within a predefined frequency band. The selection of window width must preserve the features in the signal, that is, it must correspond to the frequency band used. For narrow band signals the window is determined by the lower frequency limit since this defines what brain rhythm (synchronized oscillations) survive the filtering process. In the narrow band computations we will describe next the “cycle-criterion” (CC), as defined by Cohen [28]. According to this criterion the time-window width is CC = 2 cycles of the lower frequency. For the time step we used 5 samples, moving forward by that amount the centre of the window and recomputing the various quantities for the whole network connectivity based on the new signal segments. 

#### 2.1.2. Functional Connectivity Graphs (FCG)

To detect and precisely characterize neural synchrony between distinct recording sites, one can employ various synchrony measures like the phase locking value (PLV) [29], phase lag index (PLI) [30], coherence, and mutual information over the corresponding signals. These measures are applied, using *N* signals filtered within a particular frequency band, to every possible pair of electrodes or regions of interests (ROIs). The derived quantities are tabulated in an [*N* × *N*] matrix in which an entry conveys the strength of the functional connection between a particular pair. This matrix has a natural graph representation, called hereafter the “functional connectivity graph” (FCG), with the nodes being the recording sites and edges representing the in-between links weighted by the tabulated value. In this study we put emphasis on phase synchrony and decided to experiment with two different measures of phase coupling, namely, PLV and PLI. In our multitrial setting, both measures have been adapted so as to describe response related synchronization (as a putative mechanism for long-range integration) during information flow in visual cortex.

Phase synchrony measures have recently gained great popularity as tools for the study of brain network organization. Based on the theoretical argument that weak coupling first affects the phases of oscillators, the detection of phase synchronization is considered sufficient to reveal interactions between two weakly coupled (sub)systems [31]. In addition, brain signals and phase synchronization in specific frequency bands are thought to play a critical role in neuronal information processing [32]. Recent studies have demonstrated the pivotal role of phase synchronization in memory processes [33] and mental calculations [34]. 

PLV is the basic measure for frequency-specific synchronization between two signals. For every trial *k*  (*k* = 1,…, *N*_trials_), and for every latency *n*, the instantaneous phase *φ*(*n*, *k*) is extracted using the signal segment enclosed within a window of  *W*_*L*_  samples long. The window length is defined, independently for each frequency band under study, as *W*_*L*_ = (CC/*f*_low_)∗*f*_*s*_ + 1, where CC denotes the number of samples, which in our case corresponds to 2 cycles of a cosine with the lowest frequency *f*_low_ in the particular frequency band, and *f*_*s*_ is the sampling frequency. For a given pair (*u*, *v*) of ROIs, the following formula estimates the latency dependent phase synchronization according to PLV estimator:

(1) PLV(u,v)(n)across trials  =1Ntrials|∑k=1Ntrials1WL ∑n′=n−WL/2n+WL/2exp⁡⁡(iΔφ(n′,k))|,

where  Δ*φ*(*n*, *k*)  is the phase difference *φ* ^*u*^(*n*, *k*) − *φ* ^*v*^(*n*, *k*) between the corresponding signals (filtered within a particular frequency range). PLV quantifies the intertrial variability of this phase difference at latency *n*. If the phase difference varies little across the trials, PLV is close to 1; otherwise it is close to 0 [29]. Usually, the above quantity is integrated over successive latencies (that correspond to the moving window) so as to achieve a more robust measurement. In our implementation, the reconstructed regional activations are filtered within known frequency bands (e.g., *α*-waves, *γ*-oscillations) and the estimated latency-dependent PLV values are used to derive the final weights for the FCGs with the statistical procedure described at the end of this section. 

PLI has been introduced as an alternative phase synchronization measure, with the additional advantage of providing coupling measurements that are insensitive to the presence of common sources or volume conduction (and/or active reference electrodes in the case of EEG). The principal idea behind PLI is to quantify, only, the (relative) phase distribution's *asymmetry*. In our latency-dependent implementation, this phase synchronization estimator takes the following form:

(2) PLI(u,v)(n)across trials  =1Ntrials|∑k=1Ntrials1WL ∑n′=n−WL/2n+WL/2|sign⁡[sin⁡Δφ(n′)]||.

The PLI is bounded 0 ≤ PLI ≤ 1 and a PLI of zero indicates either no coupling or coupling with a phase difference centred on 0 mod  *π*. Since the neuroelectric recordings take place at the quasistatic range of frequencies, volume conduction introduces a zero lag phase, which is eliminated by PLI. Depending on what frequency is used, PLI will also eliminate contributions from synchronies with short delays.

It is a common practice to trim the initial estimates of functional connectivity so as to null out insignificant couplings that always appear due to random fluctuations in any time series. Based on a Rayleigh test for the uniformity of a synchronization measure (SM), we calculated the significance of each value (significance is calculated as *p* = exp⁡⁡(−*N*_trials_∗SM^2^) [35]; with SM denoting either PLV or PLI). To correct for multiple testing, the false discovery rate (FDR) method was adopted [36]. A threshold of significance was set such that the expected fraction of false positives was restricted to *q* ≤ 0.001. The PLV^(*u*,*v*)^ (or PLI^(*u*,*v*)^) values surviving this threshold were used to fill the weighted adjacency matrix  **W**  (the tabular counterpart of a given FCG).

#### 2.1.3. Characterizing FCGs via Network Metrics

FCGs can be described [37], classified, and selected as representatives of a group [38] according to various network metrics. Here, we characterize FCGs using a popular topological metric established for weighted connectivity graphs known as local efficiency (LE) [39]. It is defined as

(3) $$\begin{array}{c} \text{LE}=\frac{1}{N}\sum_{i\in N}\frac{\sum_{j,h\in G_{i,j,h\neq i}}^{}{\left(d_{jh}\right)}^{-1}}{k_{i}\left(k_{i}-1\right)} \end{array}$$

with *N* representing the total number of nodes in the network (i.e., the number of selected ROIs), *k*_*i*_ corresponding to the total number of neighbors of the current node, while *d* denotes the shortest absolute path length between every possible pair in the neighborhood of the current node. LE is understood as a measure of the fault tolerance of the network, indicative of how well subgraphs exchange information when the indexed node is eliminated [40]. Specifically, each node was assigned the shortest path length within the subgraph, *G*_*i*_. 

#### 2.1.4. Detecting Modules in the FCG

A recently introduced graph-theoretic algorithm [38] is employed for identifying the most-cohesive group of vertices given the undirected weighted matrix  **W**  of a graph. The algorithm is based on the identification of the dominant set of nodes and when repeatedly applied yields the effective clustering, in a sequential mode, of pairwise relational data. One of its main characteristics is the compact, elegant formulation. In our case takes the form of deriving the *N*-dimensional vector  **y**  that maximizes the following objective function (i.e., the set-compactness):

(4) max⁡⁡F(y)=yTWy,            y∈Δ,  Δn={y∈RN:yi≥0  ∀i∪∑i=1nyi=1}.

Following a random initialization (with *N* = (no. of sources) small positive numbers as the components of  **y**), a simple recursive formula leads to the desired solution:

(5) $$\begin{array}{c} y_{i}^{\left[k+1\right]}=y_{i}^{\left[k\right]}\frac{{\left(Wy^{\left[k\right]}\right)}_{i}}{{\left(y^{\left[k\right]}\right)}^{T}Wy^{\left[k\right]}},\;i=1,2,\ldots ,N. \end{array}$$

After a fixed number of iterations, the support of  **y**  (i.e., the set of indices corresponding to its nonzero components) is computed providing the set of nodes participating in the dominant graph-component. The full partition of a graph into disjoint sets of nodes is accomplished by repeating the following three steps: (i) finding the current dominant set, (ii) removing the vertices in that cluster, and (iii) iterating again on the rest of nodes.

 With the above procedure, each single FCG is segmented into distinct graph-components. Provided that internode similarity is expressing functional coupling, the clustering result can be considered as detection of independent functional modules within the network of visual areas. The end output is a *N*-tuple **c** = [*c*_1_,…, *c*_*N*_],  *c*_*i*_ ∈ *Z* (e.g.,  **c** = [1 3 2 3 ⋯ 2 1 1 1 2]) summarizing the graph-partition. Each integer is associated with an ROI and coincides with the membership label in one of the sequentially formed groups: 1 for the most prominent functional group, 2 for the second, and so forth. We adopted as cohesive index (CI) of each cluster the objective function that is maximized by the graph clustering algorithm ([Disp-formula EEq4]). CI is defined alternative to ([Disp-formula EEq4]) as

(6) $$\begin{array}{c} {\text{CI}}_{i}=\frac{1}{N_{c}}\sum_{s=1}^{N_{c}}\text{ICS}\left(s\right), \end{array}$$

where CI_*i*_ is the CI for each cluster *i*, *N*_*c*_ is the number of nodes participated in the cluster, and ICS is the total weight of within cluster connections of each node (called intracluster strength). 

### 2.2. Experiments Used

We applied the connectivity analysis methodology described above to two sets of data, both using visual stimuli presented to the lower left and right part of the visual field. The first experiment was designed to study the role of spatial attention and the main comparison of interest to us here was between responses elicited by identical stimuli in attended versus ignored conditions. The second experiment was designed to study how the nature of stimuli (letter versus pseudoletter) affected the categorization process in the brain. The categories were defined based on either shape or identity. 

#### 2.2.1. Spatial Attention Experiment

The full details of the experimental protocol, data preprocessing and source analysis are described elsewhere [6, 41]. Here we used the subset of the data where checkerboard stimuli were used to test the effect of spatial attention on visually evoked responses. Specifically, two sets of single trial data from two different experimental runs are presented here. In both sets of trials the same ellipse-shaped high-contrast checkerboard stimuli were presented in a random order, at 10° eccentricity along the 45° diagonals in lower left or right visual hemifield. Checkerboards had dimensions of 8.5° × 6.5°, a check size of 0.85° × 0.85°, and were oriented vertically, tilted at 18° or −18° angles. All stimuli were 350 ms in duration with interstimulus interval varied randomly between 600 and 1200 ms. During a run, each stimulus exemplar (different orientations) in each hemifield was presented for six times, thus total of 36 trials are used here (3 orientations (exemplars) × 6 repetitions of each exemplar × 2 presentation sides, left and right).

 Subjects were instructed to avoid any kind of eye or body movement, maintain fixation on a central cross, and respond to stimuli appearing in the target hemifield, as accurately and quickly as possible. In one run (18 trials) the target was the left hemifield, in another run the right hemifield. The effect of spatial attention is identified by comparing the brain activity elicited by same stimuli when the attention is directed to the stimulated versus opposite visual hemifield.

 Two sets of ROIs are used in the current study for the connectivity analysis. The first set includes twelve visual cortical ROIs, bilaterally in: V1, V2, V4, V5, lateral occipital (LO) cortex and fusiform gyrus (FG). These ROIs were defined by statistically comparing visually evoked neural responses in the post- versus prestimulus periods, as described in [41]. The second set includes eight ROIs in: medial precuneus, paracentral lobule, middle frontal gyrus (MFG), right MFG, and bilateral inferior parietal lobule (IPL) and precentral gyrus (preCG). These are putative ROIs involved in the control of visual attention. They were identified by comparing the prestimulus periods of tomographic estimates from all the trials where attention was directed to visual versus auditory modality (for the details on all visual and auditory attention conditions see the full description of the experiment in [41]). 

#### 2.2.2. Letters and Shapes Experiment

In this, the letters and shapes experiment, subjects performed a two-alternative forced-choice categorization task. The task was a shape (i.e., the two stimuli within a category had similar shape), an identity (i.e., the two stimuli differed in shape), or an arbitrary task. The stimuli were letters and pseudoletters presented with either a congruent or incongruent surround, and were presented to the center, lower left or lower right of the visual field. The experiment is a continuation of an earlier experiment where stimuli were presented in the left and right part of the visual field only [7]. We will report here results from the second experiment. 

Before each run subjects memorized a pair of categories, consisting of four target items each. They were labeled 1 and 2 and were presented simultaneously on the left and right side of the screen. Next, subjects practiced the task in a training session. A trial started with a cue (fixation cross, “<” or “>”) at the center of the screen signaling to the subjects in which part of the visual field the stimuli will be presented (bottom left, centre or bottom right). We instructed subjects to fixate on this cue during the entire trial. The stimulus appeared in the cued part of the visual field after a random interval (600–1200 ms), at 8 degrees eccentricity from the center of the screen for the peripheral presentations. Cue and stimulus remained on screen until the subject responded “Category 1” (by lifting the left index finger), or “Category 2” (right index finger). During training visual performance feedback was provided, but during recording runs (*n* = 16) no feedback was given. A recording run had 144 trials, that is, 6 repetitions of each of the 24 unique trials (2 letters and 2 pseudoletters, each occurring in 2 surround conditions and 3 visual field locations). 

MEG signals were recorded while subjects performed the task. We first used standard preprocessing of the MEG signal to remove environmental noise and subject artifacts (heart beats and eye blinks). We then averaged the signals for the 6 repetitions of each unique trial condition in each run and each visual field location. Finally, we applied magnetic field tomography to the averaged signal to obtain independent tomographic reconstruction of activity throughout the brain for each timeslice of the signal (every 0.8 ms), from 200 ms before to 500 ms after stimulus onset. The analysis produces for each visual field location and for each latency (step 0.8 ms) a total of 48 tomographic estimates of activity for each stimulus type (letter or pseudoletter), and a total of 32 tomographic estimates of activity for each task condition (Arbitrary “A”, Identity “I” and Shape “S”). To define ROIs, we applied statistical parametric mapping. Factorial analysis (ANOVA) quantified how the activity in each identified ROI changed over factors of task, stimulus type, surround and visual field location. 

We again use ROIs defined functionally for the connectivity analysis. For this case we include for V1 not only the ROIs defined for the stimuli we used, but also the ones defined for placements in the top part of the visual field. The key areas for the experiment are the LO and mid-FG areas and the Cuneus (Cu). In this and the previous study [7] the separate regional analysis identified the LO and FG areas to be most relevant for the processing of letters and sensitive to task demands and the Cu to play a role in anticipation of stimuli and coordinating the resource allocation for each task.

 Our analysis of the letter and shape experiments explores how the network properties evolve in the left and right hemispheres for two distinct cases. In the first case we compare the network evolution for stimuli that are either over-learnt symbols (letters) or similar in shape, that is, a pseudoletter, that has not yet acquired the letter identity; pseudoletters must be processed each time according to their shape. In the second case we compare the network evolution for three different tasks. In the first task the subject simply responds to any stimulus without the need to make any category distinction while in the other two a selection based on category membership must be made. In the second task (identity) the categories can be differentiated by identity of the members since the shapes are mixed in each category. In the last task (shape) the elements of each category can be distinguished by a prominent shape difference (e.g., members of one category have curved surface while that of the other are rectangular).

## 3. Results

### 3.1. Spatial Attention

**Figure 1:**
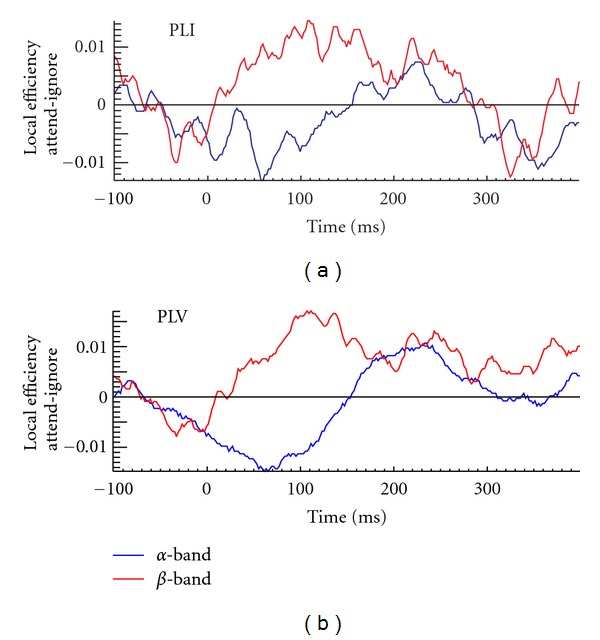
Local efficiency (LE) difference between the attended and ignored conditions as a function of latency in the *α* (blue) and *β* (red) frequency bands. These results were computed after averaging the corresponding values for the occipital sensors and for stimuli presented to the left and right bottom parts of the visual field. The results are similar for the phase lag index (PLI; (a)) and for the phase locking value (PLV; (b)). In both cases the minimum reduction in *α* band coincides with the peak of the early attentional effects in V1 (~70 ms).

**Figure 2:**
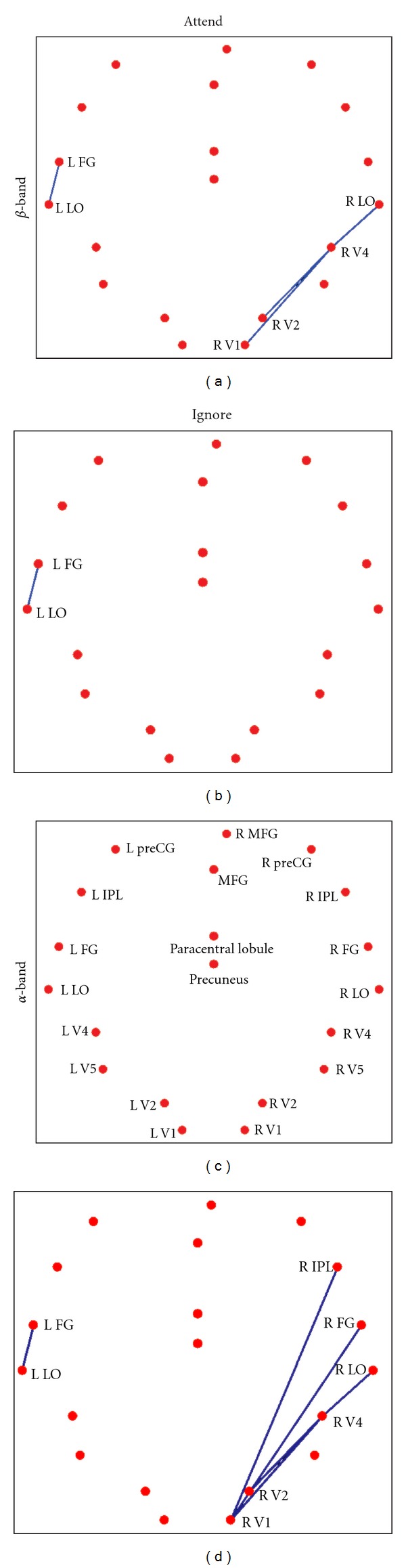
The strongest links *α* (c, d) and *β* (a, b) frequency bands for stimuli presented in the bottom left part of the visual field at 70 ms. For the *β* band the strongest links that differentiate attend versus ignore conditions are in the contralateral (right) hemisphere and connect V1 and V2 to V4 and V4 to LO. For the *α* band, the strongest links are in the ignore condition, again in the contralateral (right) hemisphere; they involve the same early visual areas, connecting V1 and V2 to V4 and V4 to LO, and in addition links between V1 and the FG and IPL.

PLV- and PLI-based FCGs were constructed for five frequency bands: *δ* (1–4 Hz), *θ* (4–8 Hz), *α* (8–13 Hz), *β* (13–30 Hz), and *γ* (30–45 Hz). We used LE of FCGs in each of these bands to identify the effect of spatial attention on event-related connectivity. The clearest attention-related changes were found in *α* and *β* bands for both PLV- and PLI-based FCGs (Figure 1). In *α*-band there was a marked attention-related decrease of LE, while opposite effect was found in *β*-band. Interestingly, these effects are identified at the same early latencies as the key regional activity modulations found in our earlier studies [6, 41]. To determine the sources of attention-related changes in LE we examined the dynamics of *α* and *β* band FCGs in attended and ignored conditions.  Figure 2 shows the PLI-based *α* (c, d)) and *β* ((a, b)) band FCGs at ~70 ms, for stimuli presented in the lower left visual quadrant, when attention was directed to the left (attended condition, (a, c)) and right (ignored condition, (b, d)) visual hemifields. In *β* band there is a strong phase coupling (increased connectivity) between early contralateral (right) visual cortical areas (V1, V2, V4, and LO) in the attended condition, which likely enables an efficient processing of attended stimuli. In the *α* band, strong phase coupling is found in the ignored condition, between the contralateral (right) visual cortical areas and the right IPL. Such coupling between the IPL, which is involved in attentional control processes [42–44], and lower level visual areas likely reflects the top-down suppression of ignored stimuli. 

### 3.2. Letters and Shapes

#### 3.2.1. Comparison between Letters and Pseudoletters

**Figure 3:**
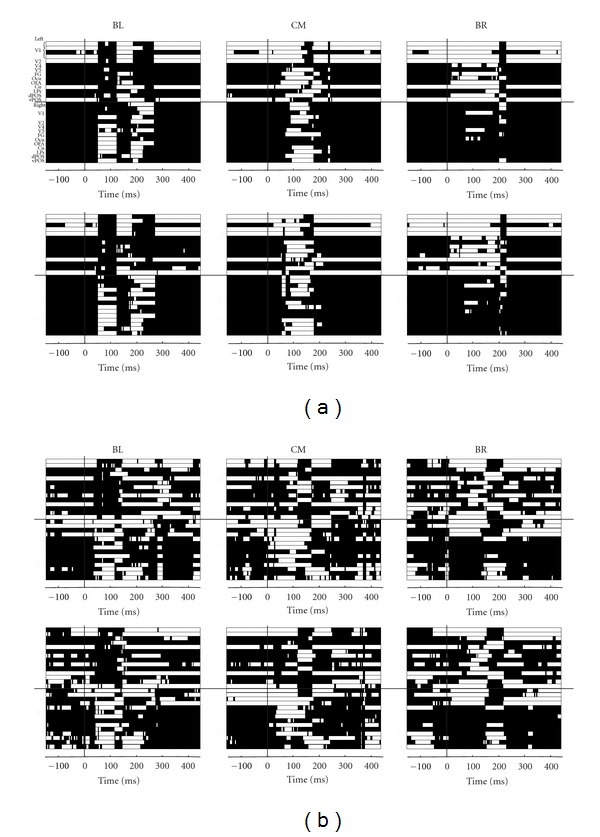
Global clustering patterns in the lower left (left column), centre (middle column), and lower right (right columns) visual fields for (a) subject 1 and (b) subject 2. Separate computations are shown for letters (upper rows in (a) and (b)) and pseudoletters (lower rows). Membership to the dominant cluster is represented by white and membership to any other cluster by black color. The computation for cluster membership was done from the wide band data pooling all three conditions (arbirtrary, identity, and shape tasks).

We first used the cluster analysis described in Section 2.1.4 to study the connectivity patterns elicited by each stimulus category (letters and pseudoletters) presented in different parts of the visual field (center, lower left, and right quadrants). The adopted clustering algorithm was applied to PLV-based FCG computed from wide band data.  Figure 3 shows the dynamics of the dominant clusters (cluster with the highest cohesive index) for two subjects.

Within each subject, the clustering patterns were different in response to stimuli presented in different parts of the visual field (compare different columns in Figure 3), as expected [25], but were very similar across different stimulus categories (compare the rows in Figures 3(a) and 3(b)). The patterns were also very different across subjects (compare Figures 3(a) and 3(b)). These results suggest existence of multiple alternative task-specific mechanisms for large-scale communication in the brain, whereas the employment of the particular mechanism is subject-specific. These results show the suitability of the method for within subject analysis while highlighting the need for care when group analysis is attempted.

 We describe in a little more detail the connectivity pattern for subject 1, who was significantly more experienced in the task. For this subject the connectivity pattern in the prestimulus period resolved into 3 to 5 clusters, but only one cluster had a high compactness and it was composed entirely of early visual areas and the cuneus, all in the left hemisphere. Within 100 ms of the stimulus arrival reorganization of connectivity has taken place with distinct patterns of connectivity emerging from the common prestimulus base for each visual field location. Within 30 milliseconds of a stimulus appearing in the left visual field the dominant cluster changes drastically with right hemisphere areas (contralateral to the stimulus) populating the dominant cluster and the left hemisphere ones relegated to the second cluster. For stimuli presented in the right visual field the transition is smoother as more extrastriate areas of the right hemisphere gain membership in the dominant cluster. Within 50 ms some visual areas in the ipsilateral hemisphere become members of the dominant cluster. The stationarity of the main cluster extends from 30 to nearly 200 ms for stimuli presented in the right visual field. For stimuli presented in the left visual field the pattern is stationary for shorter period suggesting that at least two stages of processing are involved. For stimuli presented at the center of the visual field, visual areas from both the left and the right hemispheres become members of the dominant cluster, which is more cohesive and has more members than in the other two cases.

For the second, less experienced, subject the organization and evolution of the dominant cluster was less organized. However few common features could be noted. In the first 100 ms of the poststimulus period, the dominant cluster was composed mainly of contralateral visual areas, while at later latencies (~200 ms) many areas from the ipsilateral hemispheres also became part of the dominant cluster. Importantly, similar to subject 1, the clustering patterns were similar for the two stimulus categories (letters and pseudoletters). 

**Figure 4:**
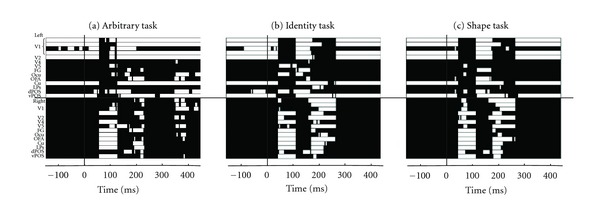
Global clustering patterns for each condition, Arbitrary (a), Identity (b) and Shape (c) for subject 1 and presentation the lower left visual field. Membership to the dominant cluster is represented by white and membership to any other cluster by black; the computation for cluster membership was done from the wide band data pooling together letter and pseudoletter stimuli presented in the lower left quadrant of the visual field.

Figure 4 shows the changes in membership of the dominant cluster (cluster with the highest cohesive index) for stimuli presented in the lower left quadrant of the visual field in the three different tasks for the first subject. Since the responses to letters and pseudoletters were very similar, we combined them within each task. There is a clear difference between the random responses (arbitrary, random response to any stimulus that appears) and the tasks where categorization must take place, either in terms of identity or the shape of the stimuli. In the ipsilateral hemisphere (left) there is only a brief change in membership within the first 100 ms, or very soon after all areas return to the dominant cluster. In the contralateral hemisphere the first response is similar in all tasks. The most noticeable difference is that for the arbitrary task, V2 and V4 are either absent from the dominant cluster, or they participate for shorter periods compared to the other two tasks. This probably reflects the reduced demand for detailed visual stimulus processing in the arbrirtrary task. Overall, in the second phase of processing the participation of most visual areas in the dominant cluster is weaker for the arbitrary task. 

**Figure 5:**
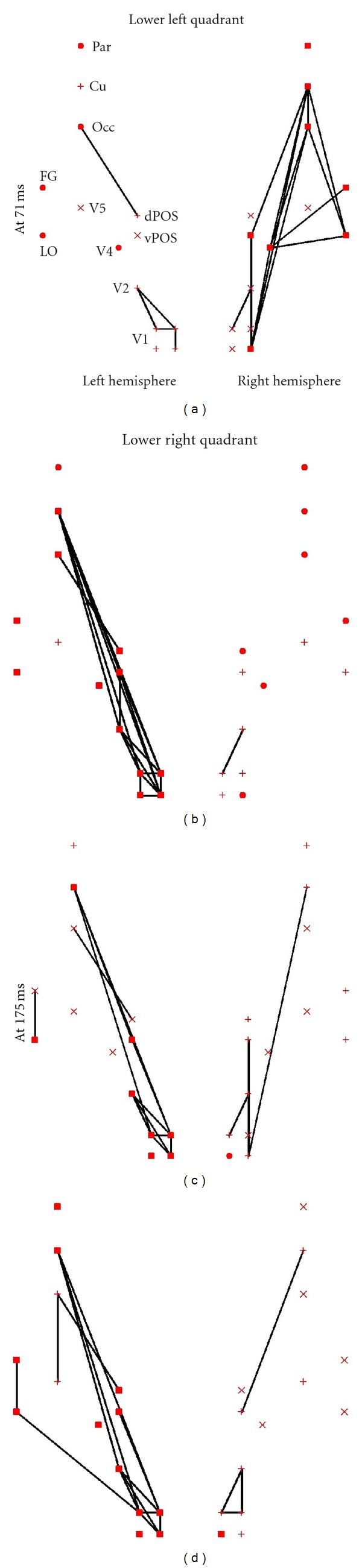
The strongest links between ROIs elicited by letters, computed from the wide band data with window center at 71 ms (top part of (a) and (b)) and 175 ms (bottom part of (c) and (d)). The results are shown separately for stimuli presented in the lower left (left part of (a) and (c)) and lower right (right part of (b) and (d)) quadrants of the visual field. Only the top 5% links are displayed that have also passed the threshold of FDR < 0.001. The results obtained from one subject are shown.

**Figure 6:**
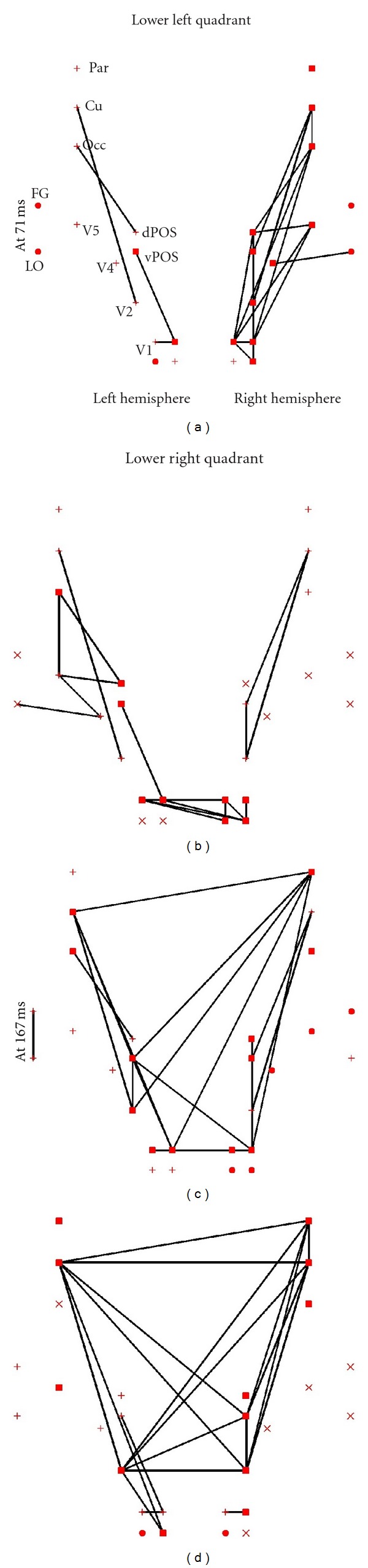
The same as in Figure 5, but the results obtained from the second subject are shown.

**Figure 7:**
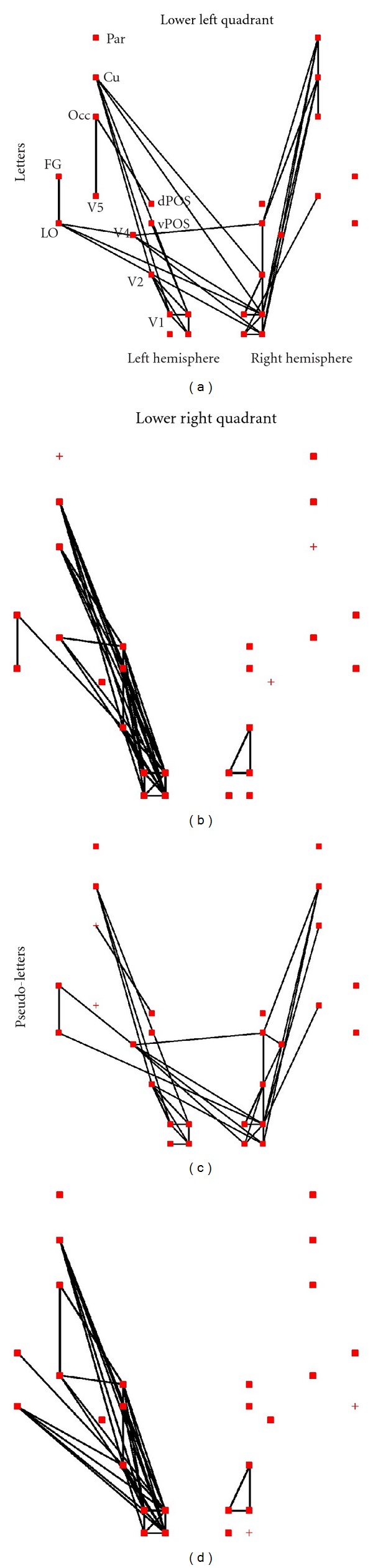
The strongest links between ROIs computed from the *γ*-band data with window center at 71 ms. The results for letters are shown in the top part of (a) and (b), and the results for pseudoletters on the bottom part of (c) and (d). The results are shown separately for stimuli presented in the lower left (left part of (a) and (c)) and lower right (right part of (b) and (d)) quadrants of the visual field. Only the top 10% links are displayed that have also passed the threshold of FDR < 0.001.

**Figure 8:**
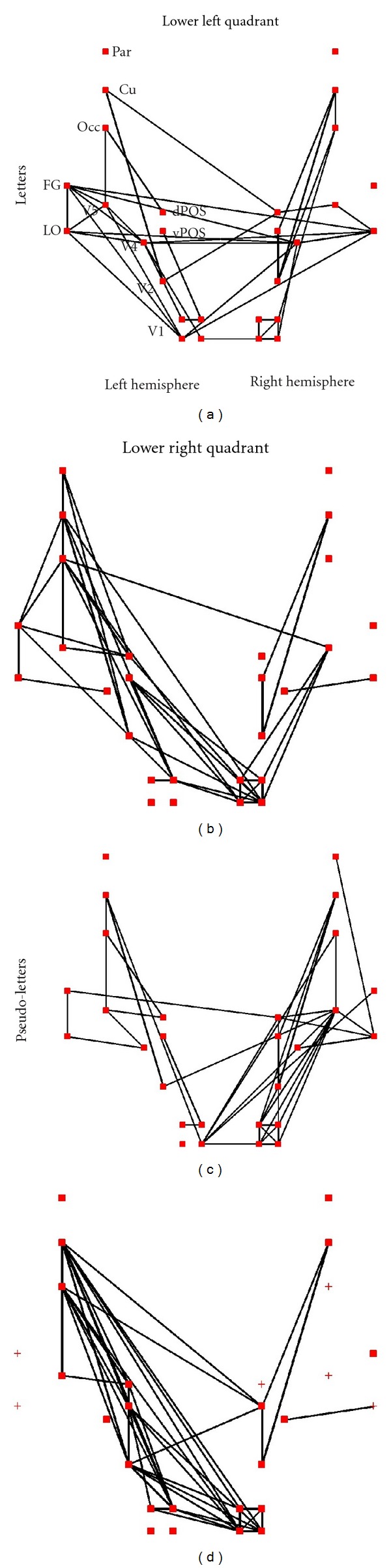
The same as in Figure 7, but the results obtained from the second subject are shown.

 The presentations in terms of the dominant clusters provide a global view of the topological organization of FCG. While for each subject such presentations revealed clear differences related to the stimulated part of the visual field, they revealed only minute differences related to the stimulus category (letters and pseudoletters) and active tasks (identity and shape discrimination). Clearly much detail remains hidden from the analysis presented in Figures 3 and 4. To study the subtle differences that distinguish the processing of different stimuli we use graph displays at key latencies extracted from wide band (Figures 5 and 6) and the *γ*-band (Figures 7 and 8). In these figures, we preserve the *k* = 5% of the strongest connections that also satisfy a significance test with FDR < 0.001; membership in the dominant cluster is indicated by a filled square symbol and membership to the second, third and fourth most cohesive clusters by an “×” a “+” and filled circle.

Figures 5 and 6 show for two subjects, respectively, the connectivity patterns elicited by each stimulus category (letters and pseudoletters) at around 70 and 170 ms using the results from the tomographic analysis of the wideband MEG signals. At ~70 ms the dominant cluster (filled squares) is composed mainly of visual areas in the hemisphere contralateral to the stimulus presentation. For the first subject the separation is complete, that is, only areas of the contralateral hemisphere belong to the dominant cluster. For the second subject a small number of areas from the ipsilateral hemisphere are clustered together with the contralateral hemisphere areas. 

 At ~170 ms we see a different pattern for each subject. For the first subject, the dominant cluster is mainly in the left hemisphere independent of where the stimulus is presented; for stimuli in the right visual field the dominant cluster includes all left hemisphere areas related to visual processing and the cuneus. For stimuli presented on the left hemisphere the dominant cluster involves early visual areas and the left cuneus, while the second dominant cluster (“×”) involves all visual areas and the cuneus on the contralateral (right) hemisphere and the extrastriate visual areas V4, V5, and FG of the ipsilateral (left) hemisphere. For the second subject, at the second phase of processing (~170 ms), the dominant cluster is made up of visual areas from both hemispheres. The difference in the clustering pattern is reflected in the connections between areas. For the first subject the connections are primarily within each hemisphere, while for the second subject there are more links between areas of the left and right hemispheres.

Across both the early (~70 ms) and late (~170 ms) phases of processing the regional activity and connections of the cuneus seem to reflect the overall connectivity pattern. A strong separation of clustering and connections in one hemisphere is associated with strong connections between the cuneus of the same hemisphere and the areas of the dominant cluster. In cases where the connections and dominant cluster involve areas from both hemisphere, the cuneus of one hemisphere (mainly in the hemisphere contralateral to the stimulated visual field) has connections with areas in both hemispheres.

 Figures 7 and 8 show for the two subjects the connectivity patterns at 71 ms elicited by letters and pseudoletters in the *γ*-band (30 to 45 Hz). Since the window extends for 30 ms on either side of the centre latency, the results display the *γ*-band connectivity pattern at the earliest stages of processing; nevertheless it is clear that the processing does not fractionate into clusters, but that almost in all cases all areas belong to one cluster. For both subjects stimulation of the lower left part of the visual field produces strong links (that survive the threshold criteria) in both hemispheres with links extending across the two hemispheres. Furthermore for left visual field stimuli links in the *γ*-band involve not only the contralateral, right cuneus, but also the left hemisphere cuneus and the extrastriate areas specialized for character processing (LO and FG); these links often connect these extrastriate areas with early visual areas (V1 and V2) of the opposite hemisphere. 

 Stimuli presented in the right visual field produce strong early links in the *γ*-band almost exclusively between areas of the left (contralateral) hemisphere for subject 1. For the second subject there is a preponderance of left hemisphere areas involved in the links in the *γ*-band but with involvement of some areas from the right hemisphere, especially V1. 

 In contrast to wide band, in the *γ*-band the differences are apparent in the connectivity patterns between letters and peudoletters, even at the early latencies depicted in Figures 7 and 8. These differences become prominent after 100 ms poststimulus (data not shown). In both subjects and for all stimuli the LO and FG in the left hemisphere, that is, the two areas best known for letter processing, show prominent links in response to both letters and pseudoletters. These two left hemisphere areas are also linked to each other for all letter stimulus cases, irrespective of which hemisphere these are presented, but they are not linked for pseudoletters presented in the contralateral (right) visual field. 

## 4. Discussion

In the present study, we investigated the dynamic changes of connectivity organization and the way transient cluster formation is associated with the preparation and execution of visual tasks. The adapted techniques have been previously introduced for tracking the formation of functional clusters in an EEG resting-state paradigm [4] and during sleep [45]. Recently, a similar in spirit methodology for tracking evolving modularity was applied to data from an fMRI experiment [46] and succeeded in showing the reconfiguration of brain networks with respect to a particular learning task. Our methodology shares with few other studies the more generic perspective of dynamic changes in clustering as the relevant framework for understanding brain function and the common goal of an objective characterization of time-varying functional connectivity. In two of the earliest attempts adaptive multivariate processes were adopted for modeling connectivity signals [47] and event-related networks were characterized based on multichannel recordings from a visual stimulation paradigm [19]. In one of the most recent works, fluctuations of functional connectivity among the nodes comprising the oculomotor network were studied in both awake humans and anesthetized macaques based on BOLD signals [48]. The general trend of combining information from different modalities is having some influence in network analysis, for example for the fusion of EEG and fMRI in network space [49]. 

 The critical novelty in our current work is the use of real time, millisecond by millisecond detailed tomographic estimates of brain activity, which allowed us to describe cluster organization at the level of the key brain areas involved in the task, and with time resolution that is within the processing periods of these areas and the transit time of information between them. Specifically we demonstrated that phase couplings in *α* and *β* bands within the visual cortical network differentiate between attended and ignored stimuli. These results suggest at least two different mechanisms by which spatial attention affects the neural processing. First, because the increased *β* oscillations are associated with efficient cortical processing, the visual cortical network synchronization in this band facilitates the neural processing of attended stimuli. On the other hand, the *α* activity band can be interpreted as an indicator of cortical inhibition and therefore the increased phase coupling in this band, especially involving a key attentional control area (IPL), realizes the top-down suppression of ignored stimuli.

In our previous analysis of MEG data elicited by letter and pseudoletter stimuli we identified the cuneus and the FG as key areas [7]. The timing and nature of the cuneus activations suggested that this structure is related to visual field and task demands, in a role that combined active anticipation and specialized routing of activity in visual processing. Our connectivity analysis revealed that the contralateral cuneus was one of the best connected areas at the earliest latencies after the stimulus onset (e.g., as seen in Figure 5), fully justifying the earlier interpretation of it having an important role in specialized routing of activity during visual processing. In our previous study, the specialized involvement of the FG emerged rather late, between 150 and 350 ms after stimulus onset in the right FG, reflecting task demands, while those in the left FG between 300 and 400 ms showing selectivity for graphemes. The connectivity analysis showed that the involvement of these two areas on the left hemisphere starts much earlier, within 100 ms in the *γ*-band with stronger participation of the left FG, irrespective of the stimulated location in the visual field. 

We have presented evidence for fast reorganization of human brain networks associated with well-defined visual tasks. We have used data from two sets of experiments where the more established methodology based on the analysis of individual regional activations showed significant results across the sets of subjects studied [6, 7]. Our results show that the view obtained from the separate study of regional activations emerges from a network activity that is very rich and with subtle dependence on task and stimulus categories, the network changes become evident well before the changes in regional activities become apparent. While the common regional activations across subjects are preserved in our connectivity analysis the details in the connectivity patterns vary a lot from subject to subject, probably reflecting different mechanisms that each subject can recruit to tackle a problem. We presented results for two subjects to demonstrate the nature of both key common features and differences across subjects as these emerge from the analysis.

There are of course a number of ways to adapt the proposed methodology for group analysis. An obvious way is to first do it independently for each subject referring to the same set of ROIs for each subject (after appropriate transformation to a common source-space). The time series of clusterings will then be forced to refer to a common time-line and can therefore be easily combined using the principles of consensus clustering [50]. After alignment the clusterings (from all subjects) can be fed to a “vector-median” computation [45] and in this way the most reliable among the individual-clusterings is selected as the representative for the whole group. The alignment can be done either by using the same latency for all subjects or allowing a different latency for each subject after time dilation and stretching to fit a given scenario (defined in advance or extracted from the data). There are however serious questions to be addressed when attempting to pool the data across subjects. First the actual anatomy differs both in terms of regional location and gray matter content of individual areas and probably even more so the effectiveness of anatomical connections. In addition the influence of activity from different brain areas on the EEG and/or MEG signal varies and in the worst case scenario the activity from some areas may produce very little EEG and MEG signal. All these problems are eliminated when the comparisons are restricted within a subject, for example, by comparing different conditions for the same subject as we have done for most of the work we presented in this paper. 

Our view is that this is an area where much work is needed before reliable across subject summaries can be obtained. The methodology is therefore ideal for within subject studies and to emphasize the point we emphasized in the presentation of our results the results from the individual subject connectivity analysis. In our opinion, the main impact of the work we have outlined will be in allowing noninvasive access to the fine details of the exquisite neural codes of individual subjects. We believe that comparing conditions within a subject could well lead to novel ways of personalized monitoring of healthy brain function and the online evaluation of remediation and rehabilitation programs, for example in developmental dyslexia training and rehabilitation after stroke.
